# The assessment of fatigue and sleep quality among children and adolescents with familial Mediterranean fever: A case-control and correlation study

**DOI:** 10.1007/s00431-024-05442-5

**Published:** 2024-02-23

**Authors:** Çağla İncesu, Gülşah Kavrul Kayaalp, Fatma Gül Demirkan, Oya Köker, Figen Çakmak, Özlem Akgün, Nuray Aktay Ayaz, Rukiye Nurten Ömeroğlu

**Affiliations:** Department of Pediatric Rheumatology, Istanbul Faculty of Medicine, Istanbul University, Istanbul, Turkey

**Keywords:** Familial Mediterranean fever, Fatigue, Sleep

## Abstract

To evaluate the sleep quality and fatigue levels in children with familial Mediterranean fever (FMF) in comparison to healthy children. The Pediatric Quality of Life Multidimensional Fatigue Scale (PedsQL-MFS) and the Pittsburgh Sleep Quality Index (PSQI) were the instruments utilized to assess fatigue and sleep quality in children with FMF and controls, respectively. Spearman’s rank coefficient was decisive in determining the association between patient-reported outcome measures and disease-related features. Two hundred twenty**-**five (59.3% female) patients and 182 (51.6% female) healthy counterparts were enrolled in the study. In PSQI, where high scores indicate sleep disturbance, the median score was significantly higher in the patient group (5; 3**–**6) than the control group (3; 2**–**4) (*p* < 0.001). PEDsQL-MFS demonstrated significantly lower fatigue levels in the control group than patients (*p* = 0.01). The level of fatigue in the patient group was found to increase in correlation with sleep problems (*r*: − 0.750, *p* < 0.001). Additionally, a high correlation was present between the PSQI/PedsQL-MFS scores and the number of attacks in the last year (*r*: − 0.645, *p* < 0.001/*r*: 0.721, *p* < 0.001, respectively). There was no difference in terms of fatigue and sleep disorders between mutations (homozygous, heterozygous, or compound heterozygous) in the MEFV gene (*p* > 0.05).

*    Conclusion*: High disease activity has a significant negative impact on the sleep quality and fatigue levels of patients with FMF. This study emphasizes the importance of assessing fatigue and sleep quality with objective outcome tools periodically in FMF patients throughout the disease course.

**Table:** 

**What is Known:** *• Fatigue is a common matter that often accompanies rheumatic diseases and causes disability*. *• Chronic rheumatic diseases often experience poor sleep quality*.	
**What is New:** *• In high correlation with the disease severity of familial Mediterranean fever, sleep quality decreases and fatigue level increases significantly*. • *In familial Mediterranean fever patients, a negative correlation is present between age and the general fatigue and sleep/rest related fatigue scores (low scores indicating greater fatigue) and sleep quality is poorer in the adolescent age group*.

**What is Known:**

*• Fatigue is a common matter that often accompanies rheumatic diseases and causes disability*.

*• Chronic rheumatic diseases often experience poor sleep quality*.

**What is New:**

*• In high correlation with the disease severity of familial Mediterranean fever, sleep quality decreases and fatigue level increases significantly*.

• *In familial Mediterranean fever patients, a negative correlation is present between age and the general fatigue and sleep/rest related fatigue scores (low scores indicating greater fatigue) and sleep quality is poorer in the adolescent age group*.

## Introduction

Familial Mediterranean fever (FMF) is the most common, inherited autoinflammatory disease characterized by recurrent episodes of fever and serosal inflammation [1]. The lifelong course of the disease can have detrimental effects on all aspects of patients’ quality of life (QoL). Patients with poorly controlled or refractory disease are at risk of impairment of both of their physical and psychosocial health [2].

Sleep is a fundamental human requirement and a key factor in enabling individuals to have a high-quality life. Adequate sleep contributes to the maintenance of both physical and mental health, as well as overall well-being. Besides, there is evidence that sleep plays a role in the defense mechanisms of the body against inflammation [3, 4]. There have been previous reports exploring the presumptive relations between the activity of inflammatory conditions and disturbances of mood and sleep. They revealed that patients with chronic rheumatic diseases often experience poor sleep quality, which can lead to various negative effects such as low academic performance, distraction, anxiety, depression, hallucinations, heightened sensitivity to pain, and loss of appetite [5–9].

Fatigue is a common matter that often accompanies rheumatic diseases and causes disability [10]. The prevalence rates of clinically significant fatigue among individuals with musculoskeletal diseases exhibit substantial variation, ranging from 35 to 80% [11–14]. Although many patients feel that fatigue surpasses pain as a source of disability, it is usually neglected and overlooked due to subjective character. In FMF, it has an unfavorable impact on the QoL and increases the burden of the disease, making the management of FMF more challenging [2, 10, 13, 15]. Data on the severity and prevalence of fatigue in rheumatic diseases are limited. Further organized studies are needed to elucidate the pathogenesis of fatigue and determine the role of biological mechanisms such as hormonal changes, inflammation, and the autonomic nervous system.

In this study, we aimed to compare the sleep quality and fatigue levels of FMF patients with healthy children, as well as to determine the fatigue level. Previous reports on sleep quality and fatigue in children with FMF are very limited and mostly based on comparative studies in the literature [2, 5, 13, 15]. Outperforming others, this study examined the factors that may contribute to sleep disorders and identified the relationship between inadequate sleep and disease activity in a large cohort.

## Materials and methods

### Ethical approvement

The study protocol received approval from the local committee of Istanbul Faculty of Medicine, dated 09.01.2020, with the reference number 104048. The research adhered to the principles outlined in the Declaration of Helsinki.

### Patient group

This cross-sectional prospective study included pediatric patients, aged 7–18 years, who had been diagnosed with FMF. The diagnosis of FMF was based on the Tel Hashomer criteria [16]. Patients were under colchicine or a combination of colchicine and interleukin-1 (IL-1) inhibitor therapy for at least 6 months in the pediatric rheumatology outpatient clinic between June 2020 and October 2020.

Healthy children between the ages of 7–18 who did not have any chronic disease and applied to the general pediatrics outpatient clinic were enrolled as the control cohort. The research population (P) was calculated as at least 192 patients using a 25% margin of error and a 5% confidence level in the power analysis. The patients were classified according to their disease severity score. The target was set for the healthy controls to be at least half the number of children with FMF.

Exclusion criteria in the patient group were determined as (1) being followed up with a diagnosis of FMF for less than 6 months, (2) having a chronic disease accompanying with FMF, (3) not agreeing to participate in the study, and (4) not filling out the questionnaire appropriately.

Exclusion criteria accepted in the control group were (1) the presence of a chronic disease or medication used regularly, (2) a history of suspected hereditary periodic fever, (3) refusal to participate in the study, and (4) improper completion of the questionnaire.

Written informed consent was obtained from the parents/guardians of the two cohorts.

### Data and measurement methods

The independent variables of the study were determined as age, age at diagnosis, gender, medication used, Mediterranean fever (MEFV) gene mutation, consanguinity between the parents, family history of FMF, and the international severity score for FMF (ISSF) [17].

Sleep quality assessment of the cohort was performed with the Pittsburgh Sleep Quality Index (PSQI). PSQI is a self-report assessment test developed by Buysse et al. in 1989 that provides information about sleep quality and the type and severity of sleep disturbance in the last month. It consists of 24 questions or rated items, 19 of which are self-reported and 5 of which require secondary feedback from a roommate or bed/room partner. Poor sleep quality was considered with a global score of ≥ 5 [18, 19]. PSQI demonstrates adequate psychometric properties for use in clinical trials involving adolescents and young adults [18, 20].

The Pediatric Quality of Life Multidimensional Fatigue Scale (PedsQL-MFS), which has been used to measure fatigue in pediatric patients since 2002, was utilized to measure the fatigue perception of children and parents. PedsQL-MFS included 18 identical items providing information for the following three sub-categories: (1) general fatigue, (2) sleep/rest-related fatigue, and (3) cognitive fatigue. Throughout the evaluation process, each sub-category comprised six distinct items, each subjected to assessment on a 5-point Likert scale. This scale ranged from zero, indicating the absence of an issue during the preceding month, to four, signifying the near-constant presence of the problem over the same period. The item scores were reversed and transformed into a standardized scale spanning from 0 to 100 (0 = 100, 1 = 75, 2 = 50, 3 = 25, 4 = 0). The total score and sub-category scores were calculated by summing up item scores and dividing the outcome by the number of items. Scores range from 0 to 100, with higher scores reflecting less fatigue. It includes distinct surveys for the following age groups: Young adults (18–25 years old), teenagers (13–18 years old), children (8–12 years old), and toddlers (5–7 years old) [21].

The distinction between musculoskeletal side effects of medications including colchicine and IL-1 blockers and myalgia and weakness resulting from underlying FMF has been made as follows: Musculoskeletal findings were considered to be suggestive of a drug-related side effect if they coincided with the initiation or recent adjustment of treatment and were considered an indicator of FMF if they occurred before drug use or were persistent in the course of FMF.

### Statistical analysis

The scoring of PSQI and PedsQL-MFS questionnaires adhered to established standard scoring systems. Categorical variables were presented as numbers and percentages, while numeric variables were described using mean, median, standard deviation, interquartile range (IQR), and min-max values. The distribution of variables underwent assessment through the Kolmogorov-Smirnov and Shapiro-Wilk tests. Analysis of quantitative independent data involved the use of independent sample* t*-test, Kruskal-Wallis, and Mann-Whitney U test, whereas the Chi-square test was employed for categorical data analysis. Spearman’s rank correlation coefficient played a crucial role in determining the association between disease activity and scores related to sleep quality and fatigue, with correlation strength categorized as low (0.10–0.29), medium (0.30–0.49), or high (≥ 0.50). Statistical analyses were conducted using the SPSS 27.0 software program, and all tests were two-tailed, considering a significance threshold of *p* < 0.05.

## Results

### Demographic data

In this prospective study, 225 individuals diagnosed with FMF and 182 healthy children were enrolled. Within the patient group, girls comprised 51.6%, while in the control group, they accounted for 59.3%. The mean age of the patients was 12.1 ± 3.3 years, and for the control group, it was 12.2 ± 3.4 years. There were no statistically significant differences in age and gender distribution between the case and control groups (*p* > 0.05).

The median total ISSF score of the patient group was 2 (IQR:2). Heterozygous, compound heterozygous, and homozygous MEFV mutations were present in 38.2%, 28%, and 25.8% of the group, respectively. At least one attack was observed in 86.7% of the patients in the last year. The clinical and demographic information of the patient group is summarized in Table 1.

**Table 1:** Clinical and demographic data of FMF patients

**Characteristic**	**Value**
Age (mean ± SD)	12.1 ± 3.4
Gender (girl/boy), (*n*,%)	116/109 (51.6%48.4)
Age at diagnosis (mean, year ± SD)	6.6 ± 2.9
FMF history in the first-degree relatives (*n*,%)	121 (53.8%)
Consanguineous marriage (*n*,%)	61 (27.1%)
MEFV mutation (*n*,%) Heterozygous Compound heterozygous Homozygous	86 (38.2%) 63 (28%) 58 (25.8%)
FMF attack (*n*,%) At least one attack in the last 6 months At least one attack in the last year No attack during the last year	111 (49.3%) 84 (37.3%) 30 (13.3%)
ISSF score (median, min–max)	2 (0–5)
Medication at last visit (*n*,%) Colchicine Colchicine + anti-interleukin 1 therapy Off drug	195 (86.7%) 26 (11.5%) 4 (1.8%)

*SD* Standard Deviation, *FMF* Familial Mediterranean Fever, *MEFV* Mediterranean Fever, *ISSF* International Severity Scoring System for Familial Mediterranean Fever

### Comparison of patients and controls

Comparisons between PedsQL-MFS and PSQI indicated a notable disparity, with the patient group exhibiting significantly higher PSQI scores (*p* < 0.001) and lower PedsQL-MFS scores (*p* = 0.01). In individuals with FMF, both general and cognitive fatigue levels were markedly lower compared to their healthy counterparts. However, there was no discernible difference between the two groups concerning fatigue specifically related to sleep or rest (Table 2).

**Table 2:** Comparison of fatigue status and sleep disturbance of controls and patients

**Feature**	**Patients**	**Controls**	***p***
PSQI (median, IQR 25–75)	5 (3–6)	3 (2–4)	< 0.001*^a^
PedsQL-MFS (median, IQR 25–75)	66.7 (56.2–79.2)	72.2 (61.1–80.9)	0.010*^a^
General fatigue (median, IQR 25–75)	66.7 (54.2–83.3)	75 (58.3–83.3)	0.004*^a^
Sleep/rest-related fatigue (median, IQR 25–75)	66.7 (52.1–75)	66.7 (54.2–79.2)	0.225*^a^
Cognitive fatigue (median, IQR 25–75)	70.8 (54.2–83.3)	75 (62.5–91.7)	0.033*^a^

*IQR *Interquartile Range*, PSQI *Pittsburgh Sleep Quality Index*, PedsQL-MFS *Pediatric Quality of Life Multidimensional Fatigue Scale

^*^Statistically significant *p* < 0.05

^a^Mann-Whitney U test

### Relationships between FMF disease characteristics and fatigue-sleep scores

Correlation analysis between the scores and the characteristics of the patients revealed that there was no correlation in terms of age at diagnosis. On the other hand, older age was associated with higher PSQI scores (*r* = 0.142, *p* = 0.033) and lower PedsQL-MFS scores (*r* = 0.03, *p* =  − 0.145). As the number of attacks and therefore the severity of the disease increased, the scores of fatigue parameters were found to be significantly lower, indicating greater fatigue. A significant negative correlation was present between the PSQI score and PedsQL-MFS score, general fatigue score, sleep/rest-related fatigue score, and cognitive fatigue score (Table 3). ISSF, PSQI, PedsQL-MFS, sleep/rest-related fatigue, and cognitive fatigue scores did not differ significantly between boys and girls in the patient cohort. General fatigue score was significantly (*p* = 0.04) higher in girls than in male patients. In the analysis made according to MEFV mutation (homozygous, heterozygous, or compound heterozygous) status, scores of ISSF, PSQI, PedsQL-MFS, general fatigue, sleep/rest-related fatigue, and cognitive fatigue did not show any significant difference (*p* > 0.05).

**Table 3:** Correlation analysis between fatigue and sleep quality scores and disease parameters

	**Correlation rank**	**ISSF**	**PSQI**	**PedsQL-MFS**	**General fatigue**	**Sleep/rest fatigue**	**Cognitive fatigue**
Age	*r* *p*	0.120 0.072	0.142 0.033*	− 0.145 0.030*	− 0.137 0.039*	− 0.197 0.003*	− 0.048 0.473
Age at diagnosis	*r* *p*	0.035 0.601	0.048 0.472	− 0.038 0.576	− 0.006 0.927	− 0.100 0.136	0.029 0.666
Attack number in the last year	*r* *p*	0.652 0.000*	0.721 0.000*	− 0.645 0.000*	− 0.568 0.000*	− 0.505 0.000*	− 0.569 0.000*
ISSF	*r* *p*		0.736 0.000*	− 0.716 0.000*	− 0.602 0.000*	− 0.559 0.000*	− 0.684 0.000*
PSQI	*r* *p*			− 0.750 0.000*	− 0.623 0.000*	− 0.624 0.000*	− 0.665 0.000*
PedsQL-MFS	*r* *p*				0.866 0.000*	0.821 0.000*	0.858 0.000*

*PSQI* The Pittsburgh Sleep Quality Index, *PedsQL-MFS* Pediatric Quality of Life Multidimensional Fatigue Scale, *ISSF* International Severity Scoring System for Familial Mediterranean Fever

*Statistically significant *p*<0.05

According to disease control status, 11 cases fell under the category of moderate control, experiencing 4 to 5 attacks annually. The remaining 214 patients demonstrated effective disease management. Despite the unequal distribution of patients in both groups, those with moderate control exhibited markedly higher disease severity, more pronounced sleep disturbances, and elevated fatigue scores (*p* < 0.001) (Table 4).

**Table 4:** Disease severity, sleep, and fatigue scores according to disease control status

	**Intermediate control (*n* = 11)**	**Well-controlled (*n* = 215)**	***p***
ISSF	4 (3–4)	2 (1–3)	< 0.001*^a^
PSQI (median, IQR 25–75)	9 (7–11)	4 (3–6)	< 0.001*^a^
PedsQL-MFS (median, IQR 25–75)	40.3 (29.2–47.2)	66.7 (58.3–80.6)	< 0.001*^a^
General fatigue (median, IQR 25–75)	37.5 (25–58.3)	66.7 (58.3–83.3)	< 0.001*^a^
Sleep/rest-related fatigue (median, IQR 25–75)	37.5 (20.8–62.5)	66.7 (54.2–76)	0.004*^a^
Cognitive fatigue (median, IQR 25–75)	37.5 (20.8–48.8)	75 (58.3–83.3)	< 0.001*^a^

*IQR* Interquartile Range, *PSQI* Pittsburgh Sleep Quality Index, *PedsQL-MFS* Pediatric Quality of Life Multidimensional Fatigue Scale

^*^Statistically significant *p* < 0.05

^a^Mann–Whitney U test

## Discussion

In this study, a comparison of children with FMF and their healthy peers for the sleep quality and fatigue levels revealed that sleep disturbance was significantly more common and fatigue levels were higher in the patient cohort. In the patient cohort, in high correlation with the disease severity, sleep quality decreased and fatigue levels increased significantly. To the best of our knowledge, this study was conducted in the largest group of pediatric FMF cohort in the literature, comparing sleep problems and fatigue together with healthy children.

FMF is a chronic disease that usually manifests clinically during childhood and adolescence, so subjects will receive colchicine for life. Patients may be prone to some physical impairment during recurrent attacks and at the time of diagnosis, both the patient and parents are informed about the natural course of the disease, which may cause some stress in children and adolescents. There are studies comparing the prevalence of fatigue in people with and without a specific chronic disease, and generally, they found that fatigue is more prevalent in patients with a chronic disease[5–8, 13–15, 22].

Sleep quality is a multi-dimensional situation influenced by personal, societal, and environmental factors, and a good quality of sleep is required for well-being. Major life events, health issues, lifestyle choices, and sociodemographic factors influence it. Therefore, FMF patients with irregularly periodic attacks are prone to changes in their usual daily activities unexpectedly without any warning sign, which may lead them to a state of sleep disorders. Therefore, FMF patients who experience irregular periodic attacks are prone to unexpected changes in their usual daily activities without any warning signs, which may lead to a state of sleep disturbances. These are general hypotheses related to chronic diseases, but they still need to be clarified with larger-scale studies.

Previous reports on chronic rheumatic and autoinflammatory diseases have revealed that the health-related QoL of FMF patients may be affected negatively by sleep and mood disorders [2, 23, 24]. In 2014, a study by Makay et al. evaluated sleep problems in children with FMF for the first time [5]. They assessed sleep disturbance by utilizing the Children’s Sleep Habits Questionnaire (CSHQ) as an instrument. As per the information gleaned from this survey, elevated scores on the questionnaire correspond to a higher frequency of sleep disturbances. The findings in the report unveiled that, despite comparable sleep durations between patients with FMF and the control group, the total sleep scores for the patient cohort were markedly higher than those of the control group. Significantly, higher scores were present in terms of sleep delay, sleep anxiety, night awakenings, and sleep-disordered breathing compared to healthy controls. A positive correlation was present between the number of attacks per year and delay in falling asleep, night awakenings, and sleep-disordered breathing [5]. Consistent with this study, our study showed that sleep problems are more common in FMF patients, with higher PSQI scores. Besides, sleep problems worsen with increased fatigue, disease severity, and number of attacks during the disease course. In another study, Küçükşahin and colleagues evaluated the relationship between sleep quality and fatigue, depression, anxiety, and disease activity in adults with FMF [24]. Their findings indicated that patients with FMF reported notably elevated scores in depression, anxiety, fatigue, and PSQI compared to the healthy control group. Their research also demonstrated a connection between poor sleep quality and levels of inflammatory markers during attacks, as well as associations with fatigue and the frequency of attacks over the last 3 months. In line with our study, they observed that as the severity of the disease heightened, both total PSQI scores and scores within its specific domains increased.

Presence of sleep disorders can lead to behavioral problems and depression in all populations. Depression, which is a common comorbidity in FMF and chronic diseases, has been associated with both fatigue and sleep disturbance and/or deficit. In the study conducted by Fredriksen et al. in 2004 on 2259 adolescents [25], it was aimed to track the effects of sleep loss in adolescents during their middle school years. Children who slept less and had sleep problems had more frequent mood and anxiety disorders. Those students who experienced reduced sleep duration in their sixth-grade year displayed lower initial self-esteem and academic grades, alongside elevated initial levels of depressive symptoms. Likewise, students with a consistent pattern of insufficient sleep reported an increase in depressive symptoms and a decline in self-esteem over time. In the study by Hysing et al., the relationship between sleep and emotional and behavioral problems in chronic diseases was investigated. It was found that patients with chronic diseases were more likely to be delayed in falling asleep and woke up at night more frequently than healthy individuals, and emotional and behavioral disorders were more common in children with chronic diseases [23]. Sleep problems should be evaluated periodically in chronic diseases such as FMF, and recovery should be achieved by controlling the disease.

Fatigue is a common and complex phenomenon and remains a significant health problem in chronic inflammatory rheumatic diseases of both adults and children such as FMF, rheumatoid arthritis, psoriatic arthritis, or systemic lupus erythematosus. Since it directly is associated with poor QoL, it can negatively affect compliance with medications and indirectly the control of the disease [26]. In 2016, Nijhof and colleagues conducted a study to ascertain the prevalence of severe fatigue and its associated limitations among adolescents grappling with pediatric rheumatic diseases. Their findings revealed that 25% of the patients in this cohort experienced severe fatigue. The study concluded that individuals with fatigue demonstrated notably lower levels of physical functioning and a higher incidence of school absenteeism compared to those without fatigue [27]. In a similar study by Özdel et al., which included 111 patients with FMF, 54 patients with other chronic rheumatic diseases, and 79 healthy individuals, fatigue levels were compared by Checklist Individual Strength-20. While similar scores were found between FMF patients and patients with other chronic rheumatic diseases, fatigue levels were significantly higher in both groups compared to healthy individuals [13]. In line with these reports, our study revealed that disease-related factors such as disease severity and the number of attacks were also correlated with fatigue levels and less fatigue was observed in well-controlled patients. Furthermore, a negative correlation was present between age and the general fatigue and sleep/rest-related fatigue scores in the PedsQL-MFS score. Fatigue level was higher, and sleep quality was poorer in the adolescent age group.

In the study conducted by Duruöz et al. in 2017, using 4 different fatigue scales in adult patients with FMF, compared to the control group, patients had increased pain, fatigue, sleep disturbance and decreased QoL. It was speculated that fatigue levels may be related to disease severity, disease duration, and attack frequency [15]. Our results similarly revealed that disease control may have a positive influence on fatigue levels in pediatric patients with FMF.

In 2001, Ben-Cherit and colleagues linked menstrual FMF attacks to the decrease in estrogen during menstruation [28, 29]. Although we did not perform an analysis regarding menstruation and disease activity in our study, there was no significant difference in PedsQL-MFS scores between the male and female patient groups. However, general fatigue levels in girls were significantly worse than in boys.

A recent study assessing the psychometric properties of PedsQL-MFS in 71 children with FMF found no discernible differences in PedsQL-MFS scores between groups, regardless of colchicine use or the presence of the M694V mutation (*p* > 0.05) [30]. In our study, no significant difference was present between patients carrying homozygous, heterozygous, or compound MEFV gene mutation in terms of sleep quality and fatigue levels.

This study is subject to several potential limitations. Firstly, the absence of an analysis pertaining to medication compliance and the level of physical activity, which can significantly influence sleep quality, restricts the multifaceted evaluations. Additionally, employing polysomnography as an objective measure could have provided a more nuanced understanding of sleep patterns. Furthermore, the cross-sectional nature of the study implies that longitudinal investigations would be more adept at elucidating the intricate relationship between disease activity, fatigue levels, and sleep quality over time. Moreover, the failure to analyze potential alterations in sleep patterns influenced by socioeconomic factors has restricted the broader applicability of the study’s findings. However, the presence of a control group, the large size of the patient cohort, and the prospective analysis using validated scales make this study superior to previous ones.

In conclusion, this study contributes to the literature by highlighting the clinical significance of assessing and managing sleep problems, as well as fatigue levels, in children with FMF. This approach, which aims to enhance the QoL of patients, should be incorporated into routine visits.

## Data Availability

The data underlying this article will be shared on reasonable request to the corresponding author.

## Abbreviations

FMF: Familial Mediterranean fever
IL-1: Interleukin-1
ISSF: International severity score for FMF
IQR: Interquartile range
MEFV: Mediterranean fever gene
QoL: Quality of life
PSQI: The Pittsburgh Sleep Quality Index
PedsQL-MFS: The Pediatric Quality of Life Multidimensional Fatigue Scale
